# Optimum Operating Conditions for the Removal of Phosphate from Water Using of Wood-Branch Nanoparticles from *Eucalyptus camaldulensis*

**DOI:** 10.3390/ma13081851

**Published:** 2020-04-15

**Authors:** Ahmed M. Mahdy, Mohamed Z. M. Salem, Asmaa M. Ali, Hayssam M. Ali

**Affiliations:** 1 Department of Soil and Water Sciences, Faculty of Agriculture (EL-Shatby), Alexandria University, Alexandria 21545, Egypt; amahdy73@alexu.edu.eg (A.M.M.); mahamedasmaa8@gmail.com (A.M.A.); 2 Forestry and Wood Technology Department, Faculty of Agriculture (El-Shatby), Alexandria University, Alexandria 21545, Egypt; 3 Botany and Microbiology Department, College of Science, King Saud University, P.O. Box 2455, Riyadh 11451, Saudi Arabia; hayhassan@ksu.edu.sa; 4 Timber Trees Research Department, Sabahia Horticulture Research Station, Horticulture Research Institute, Agriculture Research Center, Alexandria 21526, Egypt

**Keywords:** *Eucalyptus camaldulensis*, nano-scale particles, sawdust, sorption, water, pollution, eutrophication

## Abstract

A batch bio-sorption experiment was conducted on *Eucalyptus camaldulensis* Dehnh. wood-branch in the form of woody sawdust nanoparticles (nSD-KF) to evaluate their potential efficiency as phosphate bio-sorption capacity. The operating parameters of phosphate bio-sorption including contact time, initial concentration, pH, temperature, dosage, size, competing ion, and the possible mechanisms responsible for phosphate removal from water were investigated. The nSD-KF were green-synthesized by ball mill grinder and phosphate solutions with various concentrations were performed. The results revealed that the maximum adsorption capacity (q_max_) value of nSD-KF was 50,000 µg/g. In addition, the removal efficiency of nSD-KF significantly increased with the increase of initial phosphate concentration, contact time, temperature, and dosage. However, it decreased with the increase of pH and in double-system solution with the presence of ammonium ions. At the application study, the nSD-KF successfully removed 87.82% and 92.09% of phosphate from real agricultural wastewater in a batch experiment and in a column experiment, respectively. Adsorption efficiency of nSD-KF for phosphate increased after the first and second regeneration cycles, but it decreased after the third and fourth cycles. The poor to moderate phosphate desorption from nSD-KF sorbent indicates the stability of phosphate bound to nSD-KF materials. Regardless, biodegradability of nSD-KF-loaded phosphate is possible, and it will be a good source of phosphate to a plant when added to the agricultural soil as a supplemental application of fertilizer. In conclusion, nSD-KF could be considered as a promising lignocellulosic biomaterial used for the removal of phosphate from waters as bio-sorption process.

## 1. Introduction

The adverse impacts of nutrient overloading in sensitive ecosystems are becoming increasingly noticeable with higher phosphorus contents in the agricultural wastewater [1,2]. The environmental protection and remediation of all types of water are of special concern around the world. Hence, removal of phosphate from polluted water by biodegradable sorbents and its use as a supplemental fertilizer or compost are critical issues. Thus, new methods for removal of phosphate from agricultural wastewater before discharge into water bodies are required to reduce eutrophication problem.

An efficient and promising method used to remove phosphate from water is adsorption. Low-cost, availability, and biodegradability of materials used in the removal of phosphate from waters are limiting factors [3,4]. Bio-sorption is the use of biomaterials in the removal of pollutants from agricultural wastewater, where the P-loaded bio-sorbent can be reused as compost for land application in the form of supplemental phosphorus fertilizer. For the efficient and cost-saving removal of phosphate from water, the lignocellulosic biomaterials could be used in typical or modified forms [5,6,7]. Woody sawdust is one of these lignocellulosic biomaterial used for this purpose due to its chemical constituents including easily available functional groups such as OH, COOH, and NH_2_, and other compounds such as phenols, alcohols, and aldehydes that are presented in cellulose, lignin, and hemicelluloses. These functional groups can be involved in a series of chemical reactions that are able to remove the pollutants from water [8,9].

Green-synthesized nanoparticles (NPs) (<100 nm) are promising soil amendments that enable better control over of plant nutrients or contaminants release. Due to their amorphousness, NPs have a huge reactive specific surface area [10], where tremendous quantities of pollutants can be sorbed [11]. Because of the large reactive surface area of NPs, it is hypothesized that the application of nanoscale sawdust particles to wastewater would substantially increase phosphate sorption on amorphous nanoparticles and further decrease phosphate runoff and eutrophication. Subsequently, possibilities of its use as compost or supplemental P-fertilizer could be applied to agricultural soils. Hence, it is of considerable interest to define all possible operating parameters affecting phosphate bio-sorption on sawdust NPs. Therefore, the objectives of this study were to: investigate and characterize the surface chemical properties of *Eucalyptus camaldulensis* Dehnh. wood-branch sawdust nanoparticles (nSD-KF) that are green-synthesized by mechanical ball mill; evaluate the potential of nSD-KF for phosphate bio-sorption capacity; study the operating parameters including contact time, initial concentration, pH, temperature, dosage, size, competing ion, and the possible mechanisms responsible for phosphate removal from water; to test the stability, regeneration and reusability of phosphate-loaded nSD-KF; and to test the phosphate removal efficiency of nSD-KF on real agricultural wastewater.

## 2. Materials and Methods

### 2.1. Materials Collection and Preparation

*Eucalyptus camaldulensis* Dehnh. (wood-branch) was collected from Alexandria, Egypt during pruning processes. Bark of the woody species was removed, and the wood was transferred to flakes or sawdust in a sawmill located in Alexandria, Egypt.

Stock solution of 1000 mg/L PO_4_^−3^ was prepared using KH_2_PO_4_ salt. Different concentrations of phosphate solutions were prepared freshly prior to its use.

### 2.2. Green Synthesis and Characterization of Eucalyptus camaldulensis Nanoparticles

The woody-sawdust was oven-dried at approximately between 50 to 60 °C, and then mechanically ground by electrical mortar grinder (Ball mill) to produce nanoscale sawdust particles of *E. camaldulensis* (nSD-KF) according to the method of Elkhatib et al. [12]. Briefly, the nSD-KF were synthesized by grinding of bSD-KF subsample and passed through 51-µm sieve using Fritsch Planetary Mono Mill Pulverisette 6 classic line equipped with 80-mL stainless steel grinding bowl and 150 g of 1-mm steel grinding balls.

The scanning electron microscopy (SEM), quipped with energy-dispersive X-ray spectroscopy (EDX) were used for characterization of nSD-KF. The surface structure of nSDs was explored with Fourier transform infrared spectroscopy (FTIR) to illustrate the functional groups of the nanoparticle surfaces. The specific surface area (SSA) of nanoparticles was determined using the method of Brunauer et al. [13]. All these measurements were carried out by standardized methods that have been routinely used for nanomaterial studies.

### 2.3. Investigation of the Optimum Operating Conditions through Batch Bio-Sorption Experiments

#### 2.3.1. Initial Concentration and Bio-Sorption Kinetics

Conducting bio-sorption experiments were performed by shaking 0.1 g of nSD-KF with 10 mL (1:100 ratio) of phosphate solutions with various concentrations (5, 10, 20, 40, 80, 160, and 320 mg/L). The agitation was conducted at 400 rpm for 2 h using orbital centrifuge to reach sorbate-sorbent equilibrium. At 0.5, 1, 2, 5, 10, 15, 20, 30, 40, 60, 90, and 120 min, aliquot was taken for phosphate kinetics determination, then, centrifugation and filtration processes of supernatant were done using Wattman no. 42 filter paper. The filtrate was kept at 4 °C for future analysis. At each time intervals (t), the amount of sorbed PO_4_^−3^ ions by nSD-KF was calculated by the following Equation (1):

(1) $$q=\left[\left(C_{0}{\;V}_{0})-C_{f\;}V_{f}\right)\right]÷m$$

where C_0_ and C_f_ (mg/L) are the initial and final concentrations of PO_4_^−3^, respectively. V is the volume of PO_4_^−3^ solution (L) and m is the weight of the nSD-KF (g). The concentrations of PO_4_^−3^ were determined colorimetry according to ammonium-molybdate blue method [14].

#### 2.3.2. Hot-Water Extraction, pH Measurements, and FTIR Analysis

The effect of hot water extraction method and solution pH on phosphate bio-sorption by nSD-KF was studied at various pH values ranged from 7 to 11. The desired solution pH was adjusted by diluted the concentrations of hydrochloric acid and sodium hydroxide solutions. FTIR analysis was used before and after hot water extraction of un-saturated and phosphate-saturated nSD-KF [15], to identify the possible active sites modifications occurred in nSD-KF after phosphate bio-sorption. The wavenumbers of FTIR were in the range 400–4000 cm^−1^.

#### 2.3.3. Bio-Sorbent Dose and Particle Size

To study the impact of nSD-KF dosage on phosphate bio-sorption, various bio-sorbent doses from 0.5 to 2 g were tested for 40 mg/L phosphate concentration at normal pH. The effect of the particle size on phosphate bio-sorption was examined by conducting trials on different sawdust particle sizes of bSD-KF (2 mm), µSD-KF (<51 µm), and nSD-KF (<100 nm). Phosphate at the concentration of 40 mg/L and the nSD-KF dose at 0.5 g were used during the trials.

#### 2.3.4. Phosphate Bio-Sorption in Double-System

To study the effect of ammonium on phosphate bio-sorption, single and double-systems were prepared and examined, where, the initial ammonium or phosphate concentrations was adjusted at 160 mg/L and nSD-KF dose was 0.5 g at normal pH solution. To 0.50 g of the nSD-KF at natural pH, suitable amounts (10 mL) of a 160 mg/L PO_4_^−^ or NH_4_^+^ ions were added. After centrifugation and filtration of the suspension, the filtrate was stored at 4 °C until analysis.

#### 2.3.5. Thermodynamics of Phosphate Bio-Sorption

To provide information about the effect of temperature on spontaneity of the sorption process, the standard Gibbs free energy (DG_0_, J/mol), standard enthalpy (DH_0_, J/mol) and standard entropy (DS_0_, J/(mol K)) parameters were calculated.

#### 2.3.6. Removal of Phosphate from Wastewater Effluents Using nSD-KF

##### Sample Collection

Three samples of agricultural wastewater were collected from drains around agricultural fields at Abees area in one-liter pre-cleaned Teflon bottles washed with acid and deionized (DI) water. To prevent bacterial growth, ice-packed containers were used for storing the samples after collection during transit to the laboratory. All samples were filtered through 0.45 µ membrane filters (cellulose acetate) and sulfuric acid (2 N) was added to adjust the pH to 4 and then stored at 4 °C for further analysis. The water samples were chemically characterized following the joint AWWA-APHA-WEF Standard Methods guideline [16] (Table 1).

##### Performance of nSD-KF in Removing Phosphate from Actual Agricultural Wastewater Samples

● Batch Study

Efficiency of nSD-KF for phosphate removal from typical agricultural wastewater samples was examined by conducting batch technique at laboratory temperature. Known weight of dried nSD-KF was added to 40 mL of agricultural wastewater solution of known concentration and pH. The optimized conditions obtained from pure system batch sorption were used and the mixtures were vigorously stirred using a magnetic stirrer at a constant temperature for 2 h to ensure equilibrium. At different contact times, the mixtures were centrifuged and filtered. The phosphate concentration in the filtrates was determined using the appropriate methods for phosphate. The phosphate concentration retained in the nSD-KF and uptake percentage (%) were calculated.

● Column Experiment

A column experiment was carried out to investigate the phosphate removal efficiency of nSD-KF. A glass column (Ø 2 cm, 30 cm) filled with 5 g fine sand, 2 g coarse sand, and 1 g of nSD-KF were fed with 50 mL of 166 mg/L phosphate concentration (100 mg/L added in addition to the 6 mg/L phosphate present in the real agricultural wastewater). The flow rate was controlled at about 5 mL/min. Every 10 mL of the effluent was collected and analyzed to determine the phosphate concentration. Sorption capacity (q) of the column was calculated as follows:

(2) $$q=\left[\left(C_{0\;}V_{0\;}-\sum C_{n}V_{n}\right)]÷m\right]$$

where q is the sorbed amount of phosphate/g of nSD-KF (mg/g), C_o_ is the initial concentration (mg/L), V_o_ is the total volume of the influent solution (L), C_n_ is the phosphate concentration in sample n (mg/L), and V_n_ is the volume of sample n (L), m is the amount of nSD-KF (g).

#### 2.3.7. Stability of Phosphate-Loaded nSD-KF

To test the stability of phosphate-loaded nSD-KF, desorption behavior of phosphate-loaded nSD-KF was studied. A 10.0 mL of distilled water (DW) was added to 1.0 g phosphate-loaded nSD-KF, shaken for 2 h, then filtered, and analyzed for phosphate.

#### 2.3.8. Regeneration and Reusability of nSD-KF

Regeneration of the nSD-KF spent was performed by shaking phosphate-loaded nSD-KF in a specific volume (50 mL) of hydrochloric acid (0.1 mol/L) for 2 h [18]. The sorbent was filtered and washed with DW after full desorption of the PO_4_^−3^ ions. In the subsequent trials, the regenerated nSD-KF spent was reused. Four cycles of sorption and regeneration were repeated. The percentage of recovery was calculated from initial sorbed amount and desorbed amount of phosphate. Moreover, reusability of nSD-KF was conducted by adding 1.0 g doses into 10 mL aliquots of 100 mg/L phosphate solution. After shaking the suspension for 30 min, centrifugation and filtration were done. Another 10 mL portion of PO_4_^−3^ solution was agitated with the spent of nSD-KF. After shaking time 30 min, the liquid phase was separated again. These trails were repeated five times for each phosphate solution.

### 2.4. Data and Statistical Analyses

Data were statistically analyzed using the Statistical Analysis System (Release 8.02; SAS Inst Inc., Cary, NC, USA).

## 3. Results and Discussion

### 3.1. Characterization of nSD-KF Bio-Sorbent

The X-ray diffraction (XRD) patterns of hot water extracted and not-extracted of *Eucalyptus camaldulensis* wood-branch in the form of woody sawdust nanoparticles (nSD-KF) indicated that nSD-KF sample is mainly containing high percentage of N and P. Moreover, the presence of amorphous materials was observed and this was due to the significant amount of lignin and cellulose (lignocellulosic bio-sorbent) found in nSD-KF. While, after hot water extraction, N was disappeared, but P still found (Data not shown). In addition, the presence of amorphous materials was observed due to the sample still containing a significant amount of lignin and cellulose. These results coincided with the results of Abdel-Rahman et al. [19], where the presence of lignin in sawdust sample resulted in an amount of amorphous material shown in XRD patterns.

**Figure 1:**
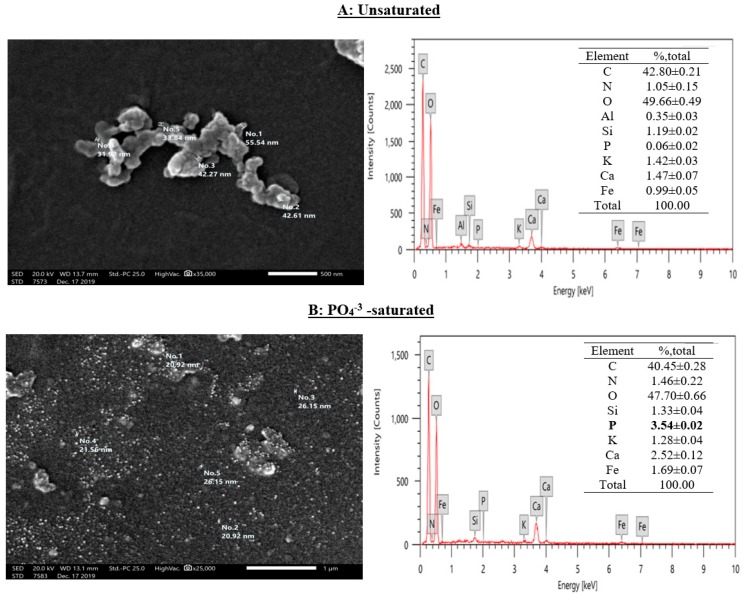
The scanning electron microscopy (SEM) image and energy dispersive X-ray elemental distribution (EDX) of un-saturated (**A**) and PO_4_^−3^-saturated (**B**)-nSD-KF.

SEM and EDX analyses of nSD-KF before and after phosphate saturation are shown in Figure 1A,B. The SEM images of nSD-KF samples clearly showed that the representative single particle size dimension lies in the range 1–100 nm. After saturation with phosphate, a layer of sorbed phosphate on the nSD-KF surface was observed which ascertained adsorption of phosphate by nSD-KF (Figure 1). The elemental analysis of nSD-KF illustrated in Figure 1 confirmed these results by appearance of phosphate (3.54%) peak in phosphate-saturated nSD-KF. Moreover, the size distribution of Zetasizer analysis refers to size average 98 nm (< 100 nm).

The specific surface area (SSA) of nSD-KF (11.33 m^2^/g) is much higher than that of the bulk bSD-KF sample (1.53 m^2^/g). Indeed, the high SSA could supply nSD-KF with highly reactive sites for PO_4_^−3^ sorption. Obviously, the untreated and un-sieved sawdust have a lower surface area (1.43 m^2^/g) than fine dusts [20,21]. In addition, other observations were recorded on different materials such as Moringa seed waste [22], where the value of specific surface area of bulk Moringa seed waste was 177 mm^2^/g. Also, SSA values of bulk Moringa seed waste were 1.79 m^2^/g and 2.97 m^2^/g, respectively [23].

The electric potential across the layer in the electric double-layer in soil called zeta potential (ZP). The value of ZP reflects the quantity of charges absorbed by the solid [24,25,26]. Based on the above concept, the ZP characteristics of an nSD-KF was studied. The results of current study showed that ZP of nSD-KF was −26.36 mV, indicating more surface charge on particles and increase of the sorptive capacity of nSD-KF.

### 3.2. Bio-Sorption Isotherm and Kinetics of Ammonium on nSD-KF

**Figure 2:**
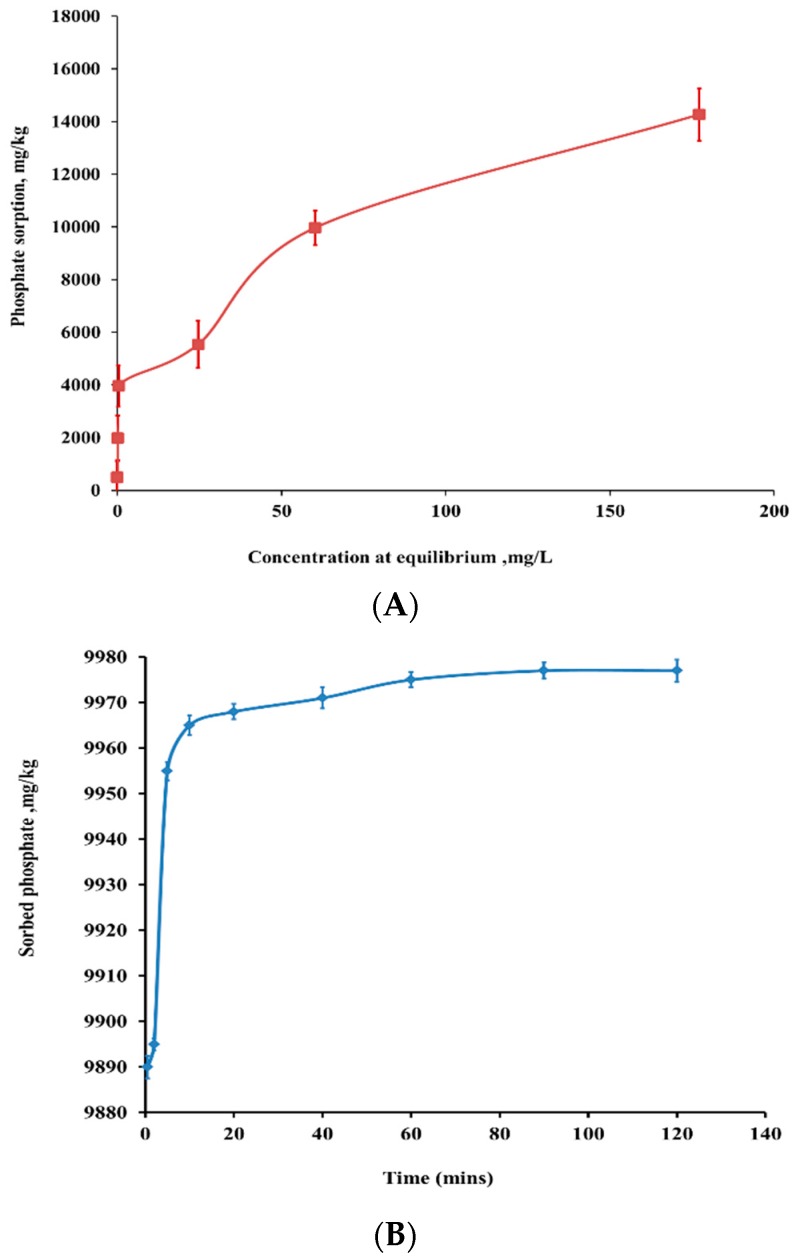
(**A**): Phosphate sorption isotherm of nSD-KF bio-sorbent, (**B**): effect of contact time on PO4^−3^ sorption by nSD-KF at initial PO4^−3^ concentration of 160 mg/L at temperature of 298 K.

Sorption isotherms were carried out to determine the sorption capacity of nSD-KF at initial concentrations range of 5 to 320 mg/L. Figure 2A shows that PO_4_^−3^ sorption by nSD-KF was as a function of PO_4_^−3^ concentration. A continuous increase of sorbed PO_4_^−3^ by nSD-KF with increasing PO_4_^−3^ concentration from 5 to 320 mg/L is noticed and the PO_4_^−3^ sorption capacity of nSD-KF was much higher. It is interesting to note that the shape of nSD-KF sorption isotherm is an ‘H’ type isotherm at low concentrations of phosphate [27], which reflects a strong interaction between PO_4_^−3^ and the nSD-KF adsorbent.

For reliable prediction of PO_4_^−3^ sorption parameters on the nSD-KF, the Langmuir sorption isotherm model was employed:

(3) $$q_{e}=q_{\max}\left(\frac{K_{L}C_{e}}{1+K_{L}C_{e}}\right)$$

where, q_e_ (mg/g) is the phosphate adsorbed per gram of nSD-KF, C_e_ (mg/L) is equilibrium phosphate concentration in solution, q_max_ (mg/g) is the maximum adsorption capacity of the nSD-KF, K_L_ (L/mg) is the constant of Langmuir.

It was demonstrated that Langmuir was successful in describing phosphate sorption data (data not shown). The maximum sorption capacity (q_max_) value of nSD-KF was 50,000 µg/g. This is as expected, due to the specific surface area of nSD-KF (11.33 m^2^/g) being higher than the specific surface area of bulk sawdust, bSD-KF (1.53 m^2^/g). High SSA of nanoparticles drastically increased the sorption capacity and surface reactivity of used bio-sorbent [28]. Therefore, modifying bulk particles to nanoparticles greatly increased its capability for phosphate removal from polluted water. Moreover, to evaluate the bio-sorption potential of the studied nSD-KF and to determine the time required for PO_4_^−3^ to reach maximum sorption on nSD-KF, the bio-sorption kinetic was performed. For 160 mg/L PO_4_^−3^ solution, it was noted that the maximum amount of PO_4_^−3^ removed by nSD-KF was 9977 µg/g, which represents removal efficiency 62.30% (Figure 2B). In the bio-sorbent studied, the bio-sorption kinetics of PO_4_^−3^ exhibited an immediate rapid sorption by which about 99.98 % of PO_4_^−3^ was biosorbed in the first 10 min and followed by slow sorption at 298 K (Figure 2B). The rapid initial adsorption of PO_4_^−3^ was due to rapid filling up of sorption sites on nSD-KF with PO_4_^−3^ ions in the initial stages and followed by a slow migration and diffusion because of the limited numbers of active sites [29]. In addition, the PO_4_^−3^ sorption kinetics by the nSD-KF was tested using first-order kinetic equation [30].

(4) $$\ln\left(q_{o}-q\right)=a-K_{a}t$$

where q is the quantity of PO_4_^3^ sorbed per gram of nSD-KF in time t, q_o_ is the quantity of PO_4_^−3^ sorbed per gram of nSD-KF at equilibrium, and k_a_ is the adsorption rate coefficient (min^−1^).

In the first order model, the “lnq” vs. “t” relationship is linear in case of conformity of sorption-desorption to first order equation. The R^2^ value of first order model was high and the SE value was quite low (data not shown). Therefore, sorption kinetics data for nSD-KF was fitted to first order equation as evidenced by higher R^2^, and lower SE value, indicating that the type of phosphate sorption mechanism could be chemical sorption [31].

### 3.3. Factor Affecting Bio-Sorption Process

#### 3.3.1. Effect of nSD-KF Dosage and Size of Particle

**Figure 3:**
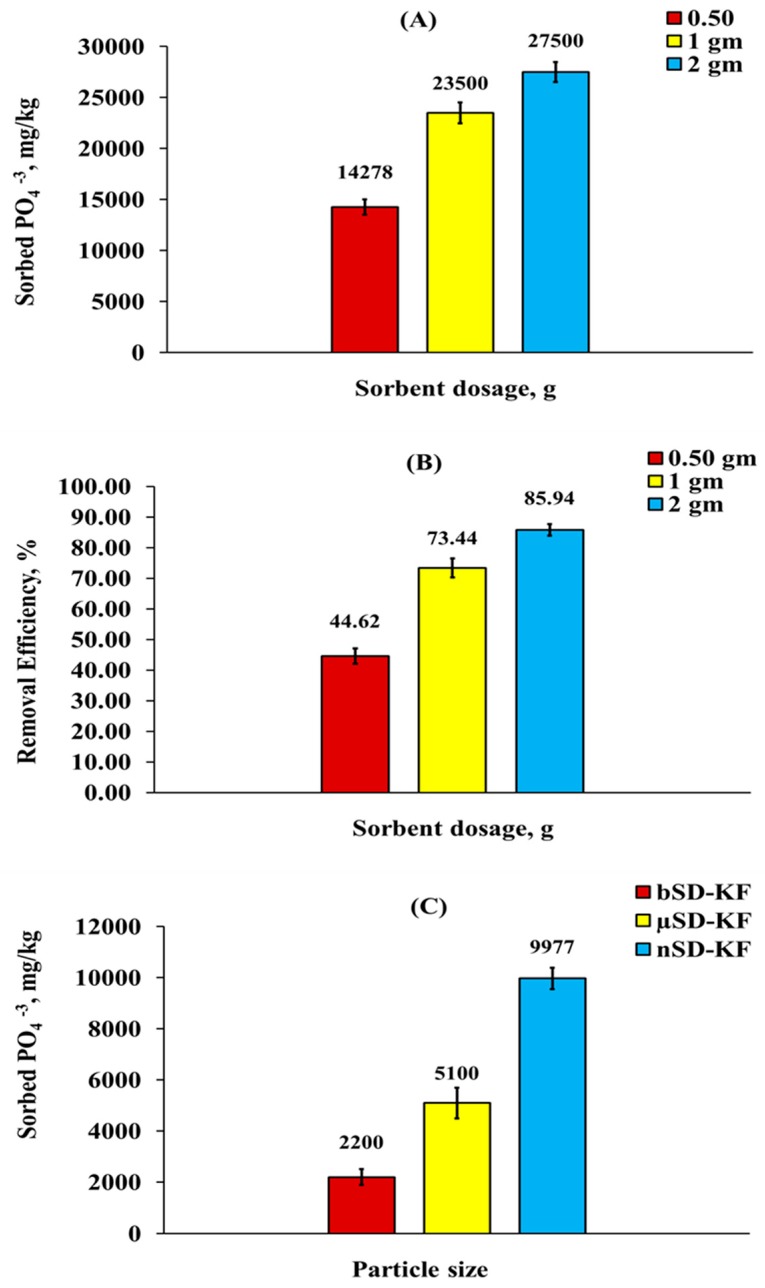
Effect of nSD-KF dose on PO4^−3^ sorption amount (**A**) and removal efficiency (**B**) from aqueous solution. (**C**): effect of particle size of sawdust on PO4^−3^ sorption amount.

The effect of nSD-KF mass on PO_4_^−3^ removal percentage was performed by adding different dosage of nSD-KF from 0.5 to 2 g using 50 mL from initial PO_4_^−3^ concentration of 320 mg/L and 120 min contact time. Figure 3A shows the effect of nSD-KF doses on the quantity of sorbed PO_4_^−3^ by sawdust particles and their removal efficiency. The results revealed that there were big variations in PO_4_^−3^ bio-sorption capacities at studied doses. By increasing the nSD-KF dosage from 0.5 to 2 g, the percentage of PO_4_^−3^ removal increased from 44.62 to 85.94 % (Figure 3B), but, the sorbed amount of PO_4_^−3^ per unit mass of bio-sorbent (bio-sorption density) decreases due to unsaturation of sorption sites during the bio-sorption process. The increase in phosphate bio-sorption with the increase of bio-sorbent dosage was due to the increase in sorption sites and SSA resulted from the increase of nSD-KF dosage, at a fixed PO_4_^−3^ concentration [32,33,34].

The effect of particle size of SD-KF on phosphate sorption was shown in Figure 3C. The amounts of sorbed phosphate were 2200, 5100, and 9977 mg/kg for bSD-KF, µSD-KF, and nSD-KF. As shown in Figure 3C, the sorption rate was significantly affected by sawdust particle size because the reduction of particle size will increase the SSA of bio-sorbent and subsequently, increase the bio-sorption chance on active sites. Besides, a little pit of the particle internal surfaces may not be utilized for sorption due to the higher mass resistance for diffusion of large size particles, and consequently the reduction in sorption efficiency [32].

#### 3.3.2. Effect of Hot Water-Extraction and Solution pH

**Figure 4:**
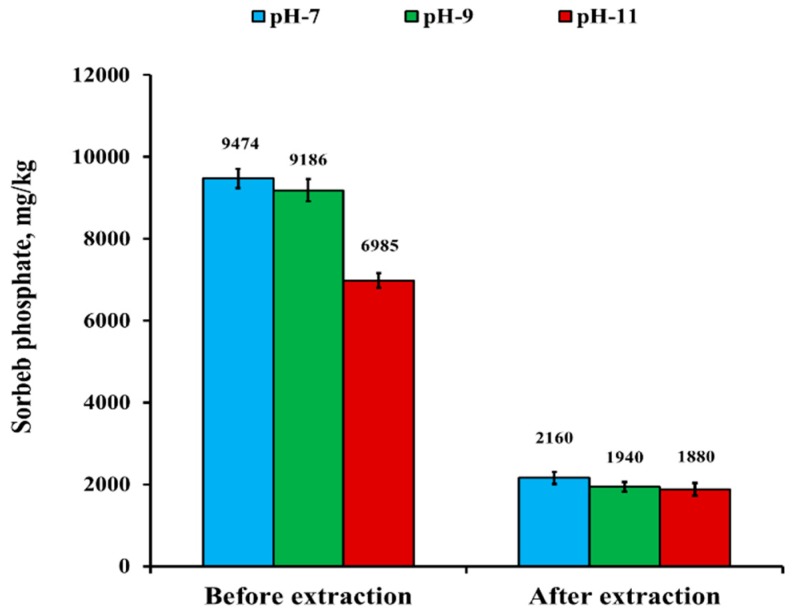
Effect of pH on PO4^−3^ sorption by nSD-KF before and after hot water extraction at the equilibrium time of 2 h and temperature of 298 K.

Due to the changes occurring in nSD-KF active sites and behavior of phosphate at different pHs [35,36,37], the effect of initial pH on the bio-sorption process was monitored in the pH range 7–11 before and after hot water-extracted nSD-KF (Figure 4) at the bio-sorbent dose of 0.1 g and initial concentration of 160 mg/L. The phosphate adsorption was highly influenced by solution pH (Figure 4). For the before hot water-extracted nSD-KF, the amount of sorbed phosphate at equilibrium was maximal (9474 mg/kg) at pH 7 and decreased to 9186 and 6985 mg/kg at pH 9 and 11, respectively. On the other hand, for the after hot water-extracted nSD-KF, the adsorbed phosphate amount at equilibrium was less than that of before-extracted nSD-KF at all measured pHs. For the hot water-extracted nSD-KF, the adsorbed phosphate amounts at equilibrium were 2160, 1940, and 1880 mg/kg at pH 7, 9, and 11, respectively (Figure 4). In comparison to the phosphate amount removed by hot water-extracted nSD-KF and the not-extracted one, the amount of phosphate removed by not-extracted nSD-KF was significantly found higher than that of extracted nSD-KF at pHs 7, 9 and 11 (Figure 4). Indeed, the amount of phosphate removed by non-extracted and extracted nSD-KF was small at pH 11 due to increasing the pH may increase the repulsive forces and competition between the negatively charged ions, causing a lower removal percentage. A similar trend has been observed for the adsorption of phosphate by biochar of sawdust [38] and water treatment residual nanoparticles (nWTRs) [12].

#### 3.3.3. Phosphate Bio-Sorption in Double-System

**Figure 5:**
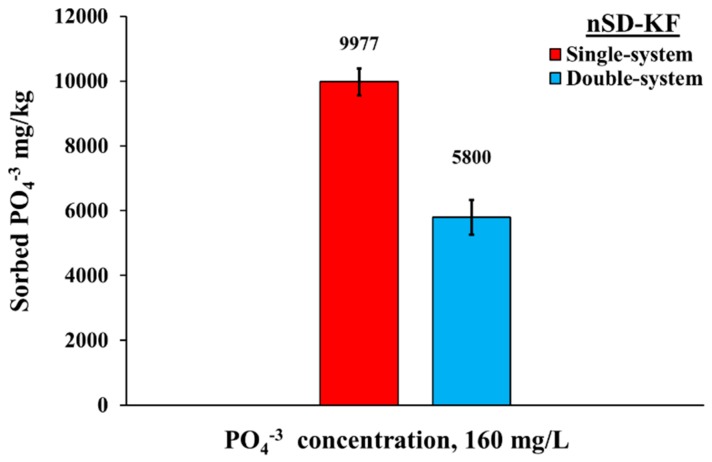
Phosphate removal in single and double-element systems by nSD-KF.

The need for recycling of N and P present in agricultural wastewater and reused as supplemental P-N-fertilization of agricultural soils is necessary due the high cost of mineral fertilizers. It is therefore important to study the phosphate adsorption onto nSD-KF biomass in presence of ammonium ions. To evaluate the phosphate sorbed by nSD-KF in single and double-element system, batch adsorption experiment was carried out in the absence and presence of ammonium at concentration equal to phosphate concentration (Figure 5). Figure 5 shows that the presence of ammonium ions reduced the amount of sorbed phosphate on the nSD-KF biomass. The percentage of phosphate removed from the single system (81.11%) by nSD-KF was higher than that removed from the double-element (NH_4_^+^ + PO_4_^−^) system (59.38%) (Figure 5) due to the adsorption possible mechanism of phosphate ions on the active sites of nSD-KF. Moreover, this decrease in double-system may be attributed to changes in the electrical double layer properties depending on the theory of surface chemistry. Similar trends were reported in other studies. For example, Ajmal et al. [39] studied the chloride effect on copper sorption and revealed that copper sorption was reduced in the presence of sodium chloride at different concentrations due to competition between Na and Cu ions. Yu et al. [8] studied the effect of acetate on the metal sorption on sawdust and concluded that there was an efficient removal of metals from acetate solution than from Cl^−^, NO_3_^−^, or SO_4_^−2^ solutions due to the inhibition of metal removal efficiency at lower pH. In addition, the results of current study agree with other observations regarding efficiency of bio-sorbent to bind heavy metals in presence of competing cations [40,41,42].

#### 3.3.4. Effect of Temperatures on PO_4_^−3^ Sorption by nSD-KF

**Figure 6:**
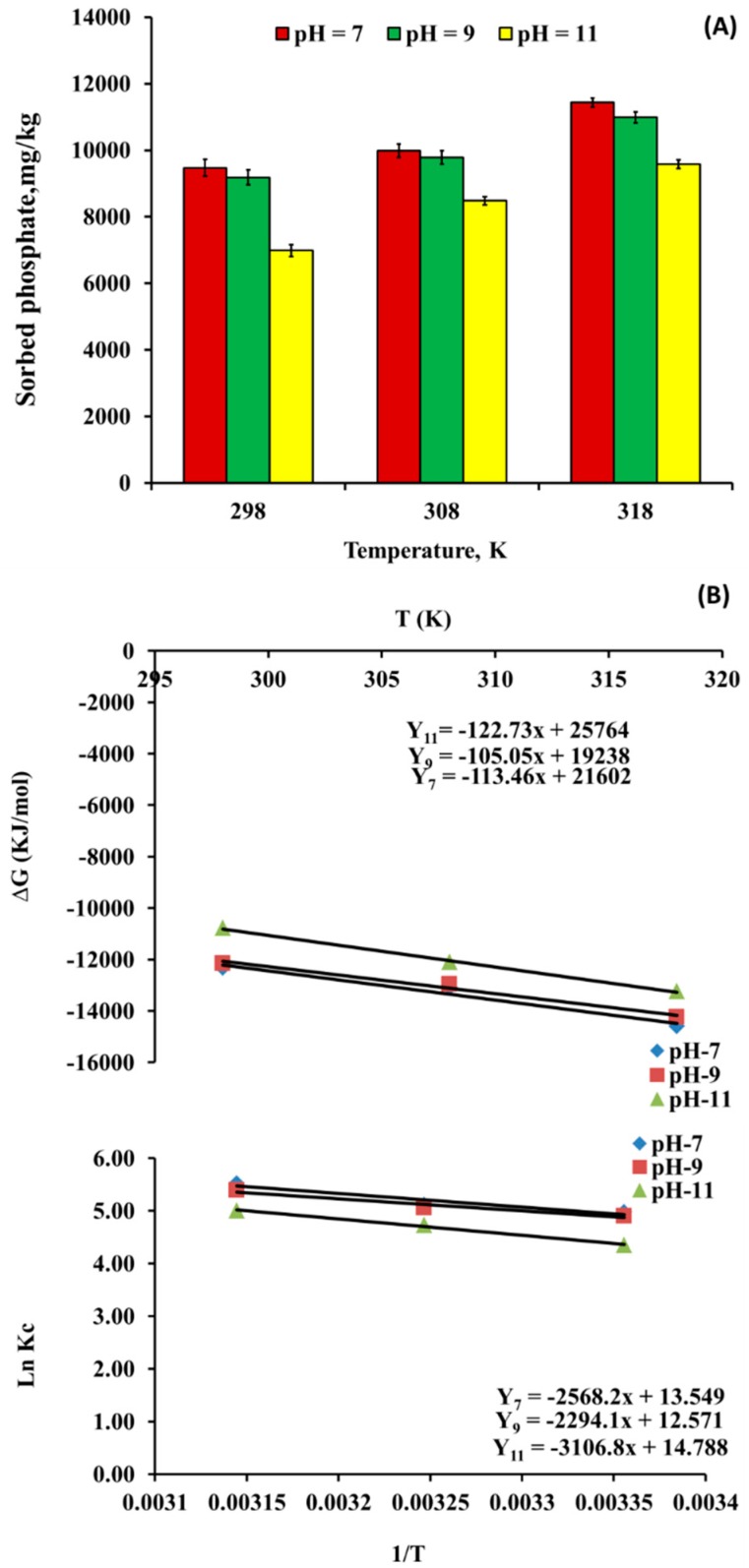
(**A**): Effect of temperature on phosphate sorption by nSD-KF. (**B**): Arrhenius plot of phosphate bio-sorption on nSD-KF. (T = 298, 308, and 318 K; pH = 7, 9, and 11; PO_4_^−3^ concentrations = 160 mg/L).

In general, phosphate bio-sorption on nSD-KF increases with the increase of temperature due to the transfer of phosphate ions to the binding sites of sawdust nanoparticles is a chemical sorption reaction [43]. The effect of temperature on PO_4_^−3^ sorption on nSD-KF bio-sorbent was studied at temperatures of 298, 308m and 318 K and 160 mg/L initial PO_4_^−3^ concentration. As shown in Figure 6A, the PO_4_^−3^ sorption increased on the studied bio-sorbent with the increase of reaction temperature from 298 to 318 K, with constant of all other reaction conditions.

At pH 7, the higher adsorption capacity was 9474, 9988, and 11,434 mg/kg at 298, 308, and 318 K, respectively (Figure 6A). The highest efficiency of PO_4_^−3^ removal by nSD-KF was at temperature of 318 K at pH 7. The increase of phosphate sorption at higher temperature may be due to cellulose structure becoming open, mobility and penetration of phosphate through sawdust may be enhanced, and the activation energy barrier will be overcame and the rate of intra-particle diffusion will be enhanced [44,45].The maximum temperature at which sorption of phosphate ions would be occurred is suggested to be 30 to 60 °C. Bryant et al. [46] pointed out that sorption of heavy metals on sawdust was significant and the solubilization of wood extractives, such as tannins, was high at 60 °C and served as the primary ion binder to wood [47]. The results of this study were in agreement with the results of Raji et al. [32] and Anirudhan and Sreedhar [48] they studied the temperature effect on heavy metal sorption by sawdust materials and reported the increase of heavy metals bio-sorption with the increase in temperature. Ismail et al. [49] studied the effect of temperature effect on cadmium ion removal by powdering corn cobs in batch experiments. They reported that there was an increase in Cd bio-sorption by the increase of temperature from 25 to 55 °C. The q_max_ of the studied bio-sorbent was observed as 18.15 mg Cd/g at 25 °C and 25.51 mg Cd/g at 55 °C.

##### Thermodynamic Parameters of Phosphate Bio-Sorption on nSD-KF

Thermodynamic parameters of phosphate bio-sorption by nSD-KF were calculated to fully understand the nature of bio-sorption. The increasing of temperature increases adsorption rate and equilibrium adsorption. The Gibbs free energy (ΔG°), enthalpy (ΔH°) and entropy (ΔS°) were determined by using following equations:

(5) $$\Delta G^{{}^{\circ}}=-{\mathrm{RTlnK}}_{c}$$

(6) $$K_{c\;}=C_{\mathrm{qe}}÷C_{s}$$

where R is the constant of gas (8.314 kJ/(mol K)), K_c_ is the constant of equilibrium, C_qe_ is the amount of sorbed NH_4_^+^ on the nSD-GU at equilibrium (mg/L), and C_S_ is the NH_4_^+^ concentration at equilibrium (mg/L).The qe of the Langmuir model was used to obtain C_qe_ and C_S_. Also, ΔG° can be calculated as follows:

(7) $$\Delta G^{{}^{\circ}}=\Delta H^{{}^{\circ}}-T\Delta S^{{}^{\circ}}$$

where ΔH° is the enthalpy change (J /mol) and Δ S° is entropy change (J/mol K). ΔH° and ΔS° can be calculated from the plot of ΔG° versus T (Figure 6B).

(8) $$L_{n}K_{c}=-\Delta G^{{}^{\circ}}÷\mathrm{RT}=\Delta S^{{}^{\circ}}÷R=\Delta H^{{}^{\circ}}÷\mathrm{RT}$$

G° or S° and H° are often calculated from a plot of ln Kc vs 1/T. The bio-sorption process will be feasible and spontaneous if the value of G° is negative. The positive value of H° indicates to endothermic sorption reaction, while the randomness at the interface between bio-sorbent surface and solution during the sorption process can be described by the S° value.

The thermodynamic parameters for phosphate sorption on the nSD-KF bio-sorbent were determined using the equilibrium constants under different experimental conditions [50]. The change in standard free energy (ΔG°) for PO_4_^−3^ sorption at initial concentration of 160 mg/L PO_4_^−3^ was observed to be −12.19, −13.32, and −14.45 kJ/mol, onto nSD-KF at 298 K, 308 K, and 318 K, respectively, at pH equal 7 (Table 2). While, the values of ΔG° were −12.07, −13.12, and −14.16 at 298 K, 308 K, and 318 K, respectively, at pH equal 9 (Table 2). Moreover, at pH equal 11, the values of ΔG° were −10.81, −12.04, and −13.27 at 298 K, 308 K, and 318 K, respectively (Table 2). The negative value of ΔG° indicates the feasibility of PO_4_^−3^ sorption process and indicates the spontaneous nature of PO_4_^−3^ sorption on nSD-KF bio-sorbent [51]. The decrease in ΔG° (i.e., increase negative) values indicated the increase in the extent of the adsorption. The decrease of ΔG° values with increasing temperature suggesting more efficient sorption at higher temperatures (Table 2 and Figure 6B) [52,53,54].

For pH 7, 9, and 11, the values of ΔS° were 0.11, 0.10, and 0.12 kJ/mol/K, respectively, for PO_4_^−3^ sorption on nSD-KF at initial solution concentration of 160 mg PO_4_^−3^/L. The positive ΔS° values indicate an increase in randomness at the solid/solution interface along with structural changes in PO_4_^−3^ and nSD-KF surface [55]. The positive ΔS° values also suggest an increase in affinity of the bio-sorbent towards PO_4_^−3^ [55]. Additionally, the magnitude and sign of ΔS° gives an indication whether the sorption reaction is an associative or dissociative mechanism. Entropy values ≥ 10 J/mol generally imply a dissociative mechanism [56]. Therefore, the values of ΔS° obtained in this study suggest that the PO_4_^−3^ sorption process involves a dissociative mechanism.

The positive values of ΔH° for PO_4_^−3^ bio-sorption on nSD-KF at initial solution concentration of 160 mg PO_4_^−3^/L (21.35–25.83 kJ mol^−1^) suggested that sorption was endothermic in nature [55] and the process is chemisorption [57] (Table 2).

#### 3.3.5. Desorption Behavior, Regeneration, and Reusability of nSD-KF Bio-Sorbent

**Figure 7:**
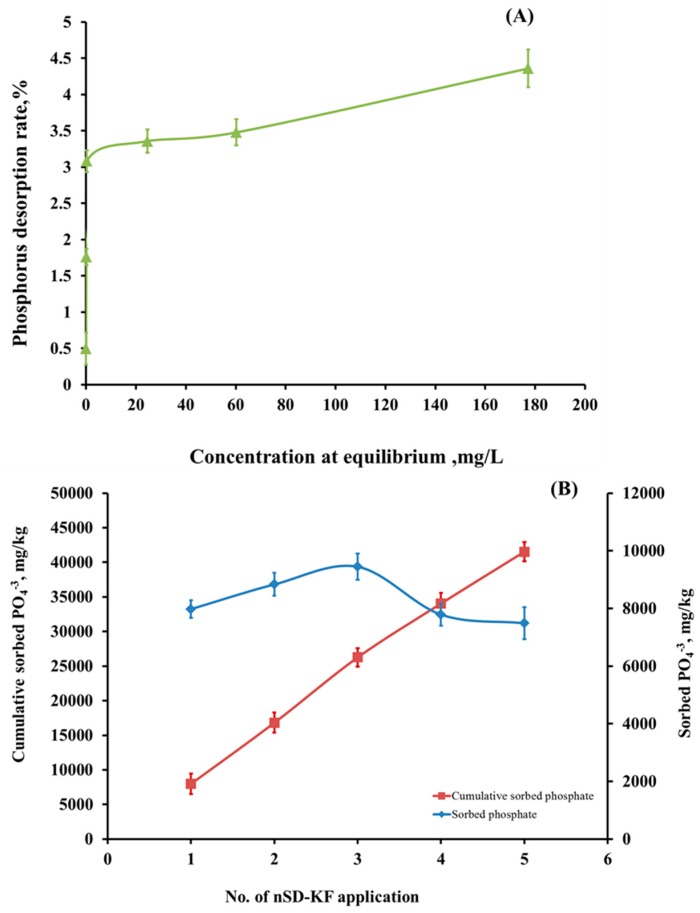
(**A**) Desorption of PO4-3 from nSD-KF loaded with different concentrations, (**B**) reusability after regeneration of the spent nSD-KF bio-sorbent (PO_4_^−3^ = 100 mg/L).

Trials were conducted to determine the desorption rate of nSD-KF sorbent (bio-sorbent reuse) (Figure 7A). As stated earlier, series of repetitive experiments were performed to assess the reusability of nSD-KF in successive adsorption-desorption cycles. The PO_4_^−3^ desorption rate after four successive cycles are shown in Figure 7A. The PO_4_^−3^ desorption rate was increased from 0.22 to 3.50% with the increase of PO_4_^−3^ concentrations 5–320 mg/L. Figure 7A shows that the PO_4_^−3^ desorption rate percentages from PO_4_^−3^-loaded nSD-KF after four successive desorption cycles was less than 4% of adsorbed PO_4_^−3^ at 320 mg/L PO_4_^−3^ concentration. The poor to moderate PO_4_^−3^ desorption from nSD-KF sorbent indicates the stability of PO_4_^−3^ bound to nSD-KF materials. Even though, biodegradability of nSD-KF-loaded phosphate is possible and it will be a good source of phosphate to plant when added to agricultural soil as a supplement fertilizer.

To assess the reusability of nSD-KF in PO_4_^−3^ adsorption, series of repetitive after regeneration experiments were performed in successive adsorption trials. The sorbed and cumulative PO_4_^−3^ sorbed by nSD-KF bio-sorbent from 100.0 mg/L PO_4_^−3^ solutions is shown in Figure 7B.

The amounts of PO_4_^−3^ adsorbed from 100 mg PO_4_^−3^/L solution were 7978, 8834, 9448, 7787, and 7489 mg/kg for adsorption cycles 1, 2, 3, 4, and 5, respectively (Figure 7B). Whereas, the amounts of cumulative sorbed PO_4_^−3^ were 7978, 16,812, 26,260, 34,047, and 41,536 mg/kg for adsorption cycles 1, 2, 3, 4, and 5, respectively (Figure 7B). Adsorption efficiency of nSD-KF for PO_4_^−3^ increased after first and second regeneration cycles but decreased after the third and fourth cycles. The capacity of nSD-KF significantly increased after two cycles of regeneration in comparison with control. This was due to the purification of sawdust nanoparticles and removal of impurities after the two cycles of regeneration. The decrease in bio-sorption capacity after the two cycles because of nonrecoverable small portion of sorbed phosphate means there was a strong interaction between ammonium and nSD-KF bio-sorbent [58,59].

#### 3.3.6. Application Study on Real Agricultural Wastewater

##### Batch Study

The efficiency of nSD-KF for phosphate removal was determined by conducting a batch experiment on real agricultural wastewater. Real agricultural wastewater (Table 1) was spiked with 160 mg/L PO_4_^−3^ (total concentration of phosphate was about 166 mg/L) and treated with nSD-KF. The results of batch study revealed that nSD-KF successfully removed 87.82% PO_4_^−3^ from real agricultural wastewater. It is noted that there was no effect of other anions found in real agricultural wastewater on the removal efficiency of nSD-KF. This was due to the competition to bind sawdust for each component in mixture depends on the ion selectivity to sawdust [58,59].

##### Backed-Column Study

**Figure 8:**
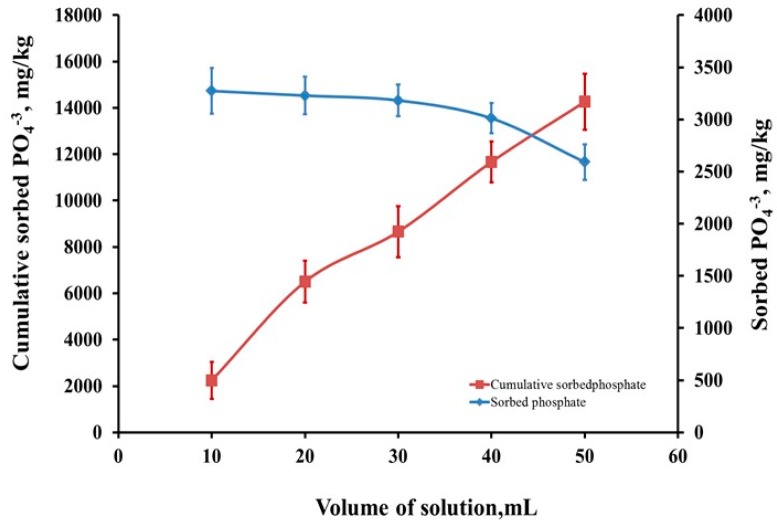
Sorbed and cumulative sorbed amounts of phosphate by nSD-KF in a packed-column study (5 g coarse sand; 5 g fine sand; 0.5 g nSD-KF; flow rate = 0.50 mL/min; PO_4_^−3^ = 166 mg/L; volume = 50 mL).

The effect of flow rate on bio-sorption of phosphate by nSD-KF was studied. The flow rate was 0.5 mL/min and keeping the initial phosphate concentration about 166 mg/L. Figure 8 revealed that phosphate concentrations was high in the first 10 mL and decreased in all volumes, but cumulative sorbed phosphate was increased with the increase of solution volumes (Figure 8). The removal efficiency of nSD-KF under continuous flow conditions using packed-column was 92.09%. Therefore, it is suggested that the obtained nSD-KF can be used as a potential sorbent for the removal of phosphate from agricultural wastewater.

#### 3.3.7. Bio-Sorption Mechanism Investigation

**Figure 9:**
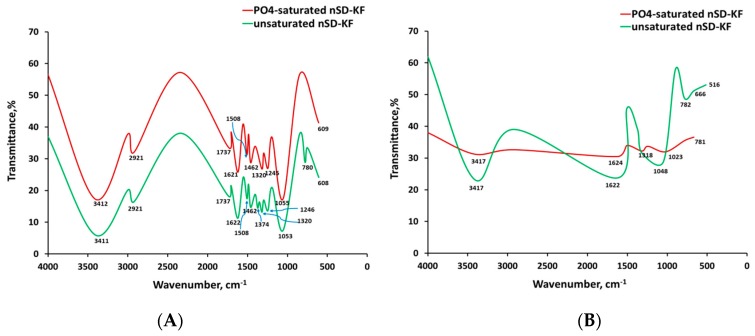
Fourier transmission infrared (FTIR) spectra of nSD-KF and phosphate-loaded nSD-KF before (**A**) and after (**B**) hot-water extraction of chemical constituents.

The FTIR spectrum of nSD-KF before and after hot water-extraction for unsaturated and phosphate-saturated nSD-KF are shown in Figure 9. The results of chemical constituents of studied nSD-KF confirmed that it is heterogeneous and this was evidenced with many functional groups found in the nSD-KF spectrum such as OH, COOH, and C=O groups. These functional groups involved in the chemical reaction between phosphate and nSD-KF as the proton donor.

To investigate the involvement of functional group found in nSD-KF, a hot-water extraction effect before and after phosphate saturation was conducted (Figure 9). The shifting and disappeared of some functional groups revealed that changes in bio-sorbent properties occurred after phosphate sorption. (Figure 9). These shifts are a strong evidence for chemical complexation between phosphate and functional groups of nSD-KF.

Before hot water extraction (Figure 9A), most changes between 1700 and 500 cm^−1^ refers to complexation of phosphate ions with COOH and O-H groups present in phenolic and carboxylic compounds [60,61,62].

After hot water extraction (Figure 9B), the FTIR spectrum before and after phosphate saturation was completely different and most changes happened between 3450 and 3200 cm^−1^, which refers to complexation of phosphate ions with OH present in alcohol and phenols of chemical compounds of nSD-KF [60].

Finally, the appearance of some minor (upward or downward) changes in spectral peaks, before and after phosphate bio-sorption on the sorbents, indicates to sorption by ion exchange [63]. The study of Volesky [64] pointed out thatbOH, C=O, and NH_2_ groups are involved in the sorption of metal. Thus, in the current study, OH, C=O and NH_2_ groups may be responsible for the bio-sorption of phosphate on nSD-KF (Figure 9).

## 4. Conclusions

The results of the current study pointed out that the removal efficiency of woody sawdust nanoparticles is very high and successfully removed 87.82% and 92.09% PO_4_^−3^ from real agricultural wastewater in the batch and column experiments and it is considered a promising lingo-cellulosic biomaterial used for the removal of phosphate from agricultural wastewaters via bio-sorption process. The removal efficiency of nSD-KF significantly increased with the increase of initial phosphate concentration, temperature, and dosage. However, it decreased with the increase of pH and in a double-system solution in the presence of ammonium ions. Adsorption efficiency of nSD-KF for phosphate increased from 7978 to 8834 and 9448 mg/kg after the first and second regeneration cycles but decreased after the third and fourth cycles. Biodegradability of nSD-KF-loaded phosphate is possible, and it will be a good source of phosphate to plant when added to agricultural soil as a supplemental P-fertilizer. The FTIR analysis confirmed that ion complexation between P and nSD-KF may occur in phosphate bio-sorption mechanism depending on the functional groups such as carboxylic, phenolic, hydroxyl, and carbonyl, which can be involved in the reactions.

## Figures and Tables

**Table 1:** The chemical analysis of the real agricultural wastewater used in the study.

Parameter	Unit	Agricultural Wastewater	IWC **
EC	dS/m	2.22 ± 0.17 *	3.00
pH	−	8.03–8.11	6.50–9.00
Cl	meq/L	11.35 ± 1.88	10.00
HCO_3_^−^	meq/L	2.13 ± 0.11	1.50
NO_3_^−^	meq/L	4.13 ± 0.44	10
PO_4_^−^	meq/L	5.61 ± 0.54	10
NH_4_^+^	meq/L	3.22 ± 0.24	10
SAR	−	7.18 ± 0.91	6–12

* Means of three samples ± SD except for pH; IWC **: Irrigation water criteria [17].

**Table 2:** Thermodynamic parameters for PO_4_^−3^ sorption by nSD-GU bio-sorbent at different solution pH values (7–11) and 160 mg L^−1^ initial PO_4_^−3^ concentration.

Initial Concentration (mg/L)	pH	T (K)	ΔG° (k J/mol)	ΔS° (k J/mol/K)	ΔH° (k J/mol)
160	7	298	−12.19	0.11	21.35
		308	−13.32		
		318	−14.45		
	9	298	−12.07	0.10	19.07
		308	−13.12		
		318	−14.16		
	11	298	−10.81	0.12	25.83
		308	−12.04		
		318	−13.27		
